# An Open Source Classifier for Bed Mattress Signal in Infant Sleep Monitoring

**DOI:** 10.3389/fnins.2020.602852

**Published:** 2021-01-14

**Authors:** Jukka Ranta, Manu Airaksinen, Turkka Kirjavainen, Sampsa Vanhatalo, Nathan J. Stevenson

**Affiliations:** ^1^Department of Clinical Neurophysiology, BABA Center, Children’s Hospital, Helsinki University Hospital and University of Helsinki, Helsinki, Finland; ^2^Department of Signal Processing and Acoustics, Aalto University, Espoo, Finland; ^3^Department of Paediatrics, Children’s Hospital Helsinki University Hospital, Helsinki, Finland; ^4^Neuroscience Center, Helsinki Institute of Life Science, University of Helsinki, Helsinki, Finland; ^5^Brain Modeling Group, QIMR Berghofer Medical Research Institute, Brisbane, QLD, Australia

**Keywords:** infant sleep, non-invasive monitoring, intensive care monitoring, NICU, bed mattress sensor, sleep-wake cycling

## Abstract

**Objective:**

To develop a non-invasive and clinically practical method for a long-term monitoring of infant sleep cycling in the intensive care unit.

**Methods:**

Forty three infant polysomnography recordings were performed at 1–18 weeks of age, including a piezo element bed mattress sensor to record respiratory and gross-body movements. The hypnogram scored from polysomnography signals was used as the ground truth in training sleep classifiers based on 20,022 epochs of movement and/or electrocardiography signals. Three classifier designs were evaluated in the detection of deep sleep (N3 state): support vector machine (SVM), Long Short-Term Memory neural network, and convolutional neural network (CNN).

**Results:**

Deep sleep was accurately identified from other states with all classifier variants. The SVM classifier based on a combination of movement and electrocardiography features had the highest performance (AUC 97.6%). A SVM classifier based on only movement features had comparable accuracy (AUC 95.0%). The feature-independent CNN resulted in roughly comparable accuracy (AUC 93.3%).

**Conclusion:**

Automated non-invasive tracking of sleep state cycling is technically feasible using measurements from a piezo element situated under a bed mattress.

**Significance:**

An open source infant deep sleep detector of this kind allows quantitative, continuous bedside assessment of infant’s sleep cycling.

## Introduction

Recent studies on sleep quality in the intensive care units have prompted interest in early sleep monitoring due to its association with general well-being and distress (van den Hoogen et al., 2017; Werth et al., 2017a). Compromised sleep in infancy is also considered to increase the risk of neurodevelopmental delay (Paruthi et al., 2016; van den Hoogen et al., 2017). Several studies have indicated that infant’s ability to fluctuate between sleep states, a.k.a. sleep-wake cycling (SWC) carries important prognostic information. Bedside tracking of SWC is still based on visual assessment of a compressed trend display of scalp-recorded electroencephalograph (Thoresen et al., 2010; Klebermass et al., 2011), which essentially identifies alternation between deep sleep (quiet sleep) and other vigilance states.

A wide range of methods have been used for the assessment of sleep in infants (Werth et al., 2017a,b). The gold standard in short-term infant sleep studies is polysomnography (PSG), a non-invasive technique that combines a large set of physiological signals, recorded overnight, to generate an assessment of sleep behavior (Grigg-Damberger et al., 2007). For long-term studies, infant sleep behavior is assessed with sleep diaries and questionnaires (Sadeh, 2004; Paavonen et al., 2019). Recent work has also used wrist- or ankle-worn actigraphy (Sadeh, 2011; Paavonen et al., 2019) to provide rough assessments of sleep-wake cycles. All of these methods have significant limitations. The use of PSG is hampered by its relative obtrusiveness and is labor-intensive in both recording and analysis, questionnaires have only limited accuracy and reliability, while actigraphy is challenged in infants due to many factors that confound interpretation (Sokoloff et al., 2020).

Several alternative solutions have been recently proposed for infant sleep studies, based on one or more physiological signals, such as cardiac, respiratory, or body movements. These works have shown clearly that wake and sleep states exhibit characteristic changes in respiration variability, body movements, and heart rate variability (Haddad et al., 1987; Harper et al., 1987). As a result, rules have been proposed for sleep scoring based on body movements and changes in the pattern of respiration, both of which can be reliably recorded with bed mattress sensors (BMS) (Thoman and Tynan, 1979; Erkinjuntti et al., 1990; Kirjavainen et al., 1996).

Recent developments in computational analyses have introduced several sleep state classifiers that are based on one or more signals in the PSG recording (Held et al., 2006; Gerla et al., 2009), such as electroencephalography (EEG) (Dereymaeker et al., 2017; Koolen et al., 2017; Ansari et al., 2020), electrocardiography (ECG) (Werth et al., 2017b) and respiratory inductive plethysmography (RIP) (Sazonova et al., 2006; Terrill et al., 2010; Terrill et al., 2012; Long et al., 2015; Isler et al., 2016). These classifiers have used heuristics (Haddad et al., 1987), computational thresholds (Terrill et al., 2012; Isler et al., 2016), discriminant analyses (Harper et al., 1987) and machine learning approaches (Sazonova et al., 2006; Koolen et al., 2017; Werth et al., 2017a; Ansari et al., 2020) to classify sleep states.

Measuring respiration with BMS may allow non-invasive long-term monitoring in the neonatal intensive care unit (NICU). In this context, the clinical need is focused on tracking cycling between deep sleep and wake—rather than an accurate detection of sleep states as in the clinical sleep medicine unit. While several BMS-based classifiers have been developed for adults in both research (Kortelainen et al., 2010; Mendez et al., 2010; Jansen and Shankar, 1993) and commercial consumer products (Tal et al., 2017; Ranta et al., 2019), there is a dearth of BMS based sleep classifiers for infants (Thoman and Glazier, 1987).

Here, we aimed to develop a BMS-based classifier for an automated assessment of deep sleep to allow observing infant’s sleep cycling. We also studied the effects of classification architecture and augmentation with the ECG on classification accuracy.

## Methods

**FIGURE 1:**
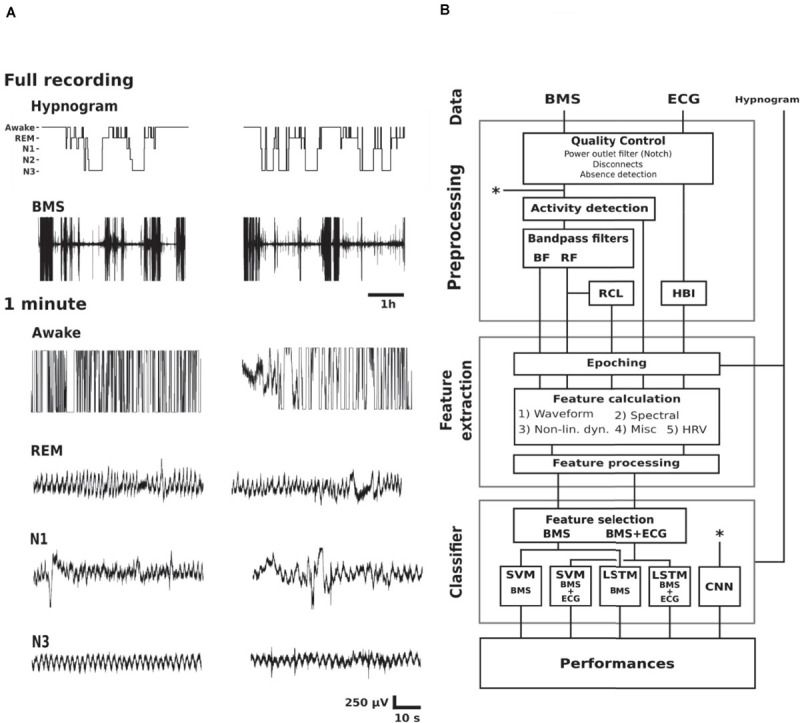
Study design. **(A)** Examples of hypnogram and BMS data of two subjects (columns) in different vigilance states. The BMS signals below the hypnograms show the full length of BMS signal where movement epochs are readily observed. The shorter 1 min epochs show examples of awake (lots of high amplitude artifacts), REM sleep (variable respiration frequency with relatively stable baseline), light sleep (N1; variable respiration frequency with baseline instability due to movements), as well as N3 (relatively stationary respiration, i.e., steady respiration frequency and amplitude). **(B)** A schematic diagram of classifier construction divided into functional blocks. Abbreviations: Rapid eye movement (REM), non-REM 1–3 (N1–3), bed mattress sensor (BMS), ballistocardiographic bandpass filtered BMS (BF), respiration frequency bandpass filtered BMS (RF), respiration cycle length series (RCL), heart beat interval series (HBI), non-linear dynamics feature category (Non-lin. dyn.), heart rate variability (HRV), support vector machine (SVM), Long Short-Term Memory neural network (LSTM), convolutional neural network (CNN). Asterisks refer to the shortcut of the CNN pipeline from the raw signal (upper asterisk) to the classifier training (lower asterisk).

This study used a retrospective collection of PSG and BMS recordings. Study design is outlined in Figure 1 including examples of BMS data in different vigilance states, and a schematic diagram of classifier construction. All classifiers were trained using labels generated by the visual interpretation of the hypnogram. The signal pre-processing, feature extraction, and selection, SVM training and testing, in addition to final analysis were conducted in Matlab [MATLAB 2016. version 9.1.0 (R2016b), The MathWorks Inc., Natick, Massachusetts]. The neural networks were trained and tested in TensorFlow (Abadi et al., 2015). The classifiers are publicly available at GitHub (Ranta, 2020).

### Data Acquisition

A data set of 51 infants was collected at the Department of Children’s Clinical Neurophysiology, Children’s Hospital, Helsinki University Hospital (see Table 1 and Supplementary Table S1), including all infants (< 6 months) that were assigned to a clinical sleep study in 2016. The PSG data was gathered using Embla N700 equipment and RemLogic 3.2.0 software (Natus, United States) as per routine clinical protocol. A thin 40 cm × 50 cm × 6.35 mm electromechanical ferroelectric sleep mattress sensor (model L-4060SLC, Emfit, Finland) was recorded as an additional channel in the analog input of the PSG recorder (pass band between 0.07 and 48 Hz). All signals were sampled at 200 Hz. The thin, cellular, quasi-piezoelectric film sensor generates a charge when the external pressure changes (Paajanen et al., 2000, 2001). The data set was visually reviewed to exclude recordings with poor signal quality, i.e., no visually detectable respiration, leaving 43 PSG recordings for classifier development. The study was approved by the Institutional Review Ethics Committee of Helsinki Hospital.

**TABLE 1:** Summary of the participants.

**Sleep summary**	**Median**	**Min**	**Max**
Age (weeks)	6	1	18
Recording time (h:min)	4:12	2:22	9:05
Total sleep time (h:min)	2:17	0:48	7:51
N3 time (h:min)	0:52	0:18	3:03
Diagnoses			# Cases
Obstructive sleep apnoea			18
Central sleep apnoea			4
Increased work of breathing			5

*The age indicates postnatal age, or age after term equivalent age.*

In addition, we tested the real-life feasibility of this method by recording two newborn infants during their stay in the NICU. These infants both represent the key target group where SWC monitoring is of interest during the early stay in the NICU (Thoresen et al., 2010; Klebermass et al., 2011): They were both born at full-term and recorded during days 2–3 after birth. One infant was admitted to NICU due to a placenta ablation during labor, leading to resuscitation. The other infant was taken to NICU due to severe birth asphyxia. No other brain pathology was known by the time of recording. The recordings included bed mattress as above, as well as a standard four electrode EEG to be inspected as the amplitude integrated trend (aEEG), the basis of current bedside detection of SWC (Thoresen et al., 2010; Klebermass et al., 2011). The SWC in the aEEG trend was visually compared to the output of BMS-based classification to verify the correspondence.

Clinical review of PSG studies included sleep scoring, i.e., generation of hypnogram, followed by analysis of respiratory events. The hypnogram was scored every 30 s according to the AASM guidelines by a clinical expert (T.K.) who was unaware of this study at the time of clinical sleep scoring (Grigg-Damberger et al., 2007).

We chose to focus on the distinction between deep sleep (N3) and other states for two reasons: First, the clinical need for online sleep monitoring is mainly to recognize sleep-wake cycles (SWC, Kidokoro et al., 2012; van den Hoogen et al., 2017), so identifying likelihood of N3 sleep would be sufficient to quantify cyclicity in sleep states (Stevenson et al., 2014). Second, our clinical experience as well as preliminary analyses showed that distinction of more superficial sleep states, including REM, from the respiratory signals may not be as accurate as needed for SWC quantification (Figure 1A). Therefore, we designed the study to classify N3 from the BMS signals alone, or by using additional input from the ECG signal available in PSG recordings. The full processing pipeline (Figure 1B) consisted of three main blocks; pre-processing, feature extraction, and sleep classifier construction. We trained and evaluated five different classifiers, four feature-based classifiers, and one classifier with representation learning capability.

### Pre-processing

A notch filter was used to remove mains artifact (50 Hz). We applied a detection for infant’s presence/absence by using simple thresholding within 6–16 Hz band power, as infants were occasionally removed from the bed. Gross-body movements and disconnects were also identified using simple thresholds applied to the smoothed root-mean-square value.

Next, we separated respiratory and cardiac activity with Butterworth bandpass filters using 0.2–1 Hz band (12–60/min respiratory rate) for respiration frequency range (RF), and 6–16 Hz band was used for identifying ballistocardiography frequency (BF). An algorithm was developed to derive respiratory cycle length time series (RCL). Since the infant heart beats were found to be too weak for robust and reliable detection from BF, we decided to extract heart beat interval series (HBI) from the R-peaks of the ECG that was part of PSG. R-peaks were identified using the Pan Tompkins algorithm (Pan and Tompkins, 1985).

### Feature Extraction

All features were computed for each 30 s epochs corresponding to the visually interpreted hypnograms. The feature set was designed to cover a wide range of physiologically reasoned features from four overall categories; waveform, spectral, non-linear dynamics, and miscellaneous. In addition, we also calculated common heart rate variability features from the ECG data (Malik, 1996). This resulted in a total of 71 BMS-derived features and 15 ECG-based features. See Table 2 for details of the feature set.

**TABLE 2:** Computational features used in the SVM and LSTM classifiers.

**#**	**Feature description**	**Abbr.**	**Source**	**References**
	*Time-domain*			
1–4	Mean	M	RCL, RF^†^, HBI	Jansen and Shankar, 1993; Long et al., 2014
5–6	Median	MED	RF^‡^	Long et al., 2014
7–12	Variance	VAR	RF^a^, RF^†^, BF^a^	Jansen and Shankar, 1993
13–19	Standard deviation	STD	RCL, RF^‡^, RF^*,a^, BF^*,a^	
20–25	Skewness	SKEW	RCL, RF^a^, BF^a^, HBI	
26–31	Kurtosis	KUR	RCL, RF^a^, BF^a^, HBI	
32–33	Coefficient of variation	COV	RCL, HBI	
34–37	Hjorth mobility	MOB	RF^a^, BF^a^	Hjorth, 1970
38–41	Hjorth complexity	COMP	RF^a^, BF^a^	Hjorth, 1970
42–53	Zero crossings	ZC	RF^d,a^, BF^d,a^	Jansen and Shankar, 1993
54	Power	POW	BMS	

	*Spectral*			
55–56	First moment	M1	RF, BF	
57–58	Second moment	M2	RF, BF	
59–60	Spectral entropy	SE	RF, BF	
61	Mode	MO	BMS	
62	Mode phase	MOF	BMS	
63	Mode width	MOW	BMS	
64	Mode power	MOP	BMS	
65	Autocorrelation function lag	ACFL	BMS	
66	Autocorrelation at lag	ACF	BMS	

	*Non-linear-dynamics*			
67–68	Sample entropy	SAMPE	RF, HBI	Richman and Moorman, 2000
69–70	Fuzzy entropy	FUZE	RF, HBI	Chen et al., 2007
71–72	Hurst exponent	HE	RF, HBI	Peng et al., 1994
73–74	Lyapunov exponent	LE	RF, HBI	Rosenstein et al., 1993

	*Miscellaneous*			
75–76	Envelope crossings	EC	RF, BF	
77	Activity duration	ACT	BMS	
78	Activity start count	ACTC	BMS	
79	Increased respiratory resistance instances	IR	RF	Alametsä et al., 2006
	*Heart rate variability*			Malik, 1996
80	Standard deviation of normal to normal R-R intervals	SDNN	HBI	
81	Root means square of successive differences	RMSSD	HBI	
82	Low-high frequency ratio	LF/HF	HBI	Mendez et al., 2009
83	Low-very Low frequency ratio	LF/VLF	HBI	Mendez et al., 2009
84	High-very low frequency ratio	HF/VLF	HBI	Mendez et al., 2009
85	High frequency mode	MODHF	HBI	Mendez et al., 2009
86	High frequency mode phase	PHF	HBI	Mendez et al., 2009

*The sources; bed mattress sensor signal in 0.07–48 Hz (BMS), respiratory frequency band 0.2–1 Hz (RF), ballistocardiography band 6–16 Hz (BF), respiratory cycle length time series (RCL) and ECG derived heart beat interval series (HBI). Feature abbreviation (Abbr) and literature reference (Ref). ^†^From extrema amplitude and time differences ^*a*^Also derived for activity events. ^†^From peaks and troughs ^*d*^Also from the first and second differential signals ^∗^ From envelope.*

Epochs judged to contain excessive movement artifacts were labeled as missing, and replaced by linear interpolation of surrounding feature values. Features with a heavy tailed distribution were log-modulus transformed (John and Draper, 1980) and a subject specific Z-score normalization was applied to reduce interpatient variability.

### Classifiers

We evaluated four feature-based classifiers using support vector machine (SVM) (Cortes and Vapnik, 1995) and Long Short-Term Memory recurrent neural network (LSTM) (Hochreiter and Schmidhuber, 1997), as well as a classifier using deep convolutional neural network (CNN) on BMS signal with a representation learning capability. For SVM and LSTM, we tested two feature sets, one with solely BMS-based features (SVM BMS, LSTM BMS) and extensions with additional ECG-based features (SVM BMS + ECG, LSTM BMS + ECG).

We deployed SVMs with radial basis kernels. In addition to treating epoch level features as discrete inputs, we also decided to build temporal context into the SVM classifier (Sazonova et al., 2006). This was reasoned by the presence of significant natural autocorrelation in hypnograms, as well as our attempt to emulate the clinical practice where sleep scores are assumed to inherit conditional inputs from the past epochs (Grigg-Damberger et al., 2007). We included feature values of 6 epochs into our classification building, thus, the classification of each 30 s epoch considers the past 3 min.

To investigate the impact of a longer temporal context we decided to use LSTM with 32 units, that takes preceding feature time series as an input. Temporally distributed layer with softmax activation function yields the class output. The network uses the same features as selected with the SVMs.

We also used an end-to-end CNN classifier, which is capable of feature representation learning from raw input data. The architecture of the utilized CNN is presented in Supplementary Table S2.

### Training and Testing

The SVM classifier was trained with a feature selection stage to understand which features are important for classification and reduce the number of unhelpful features. We used a two-stage feature selection algorithm relying on minimal-redundancy maximal-relevance criterion (mRMR) followed by a forward selection procedure (Peng et al., 2005). The kernel scale and box constraint hyperparameters were tuned within each training iteration using three-fold cross-validation and Bayesian optimization (Snoek et al., 2012).

The CNN and LSTM were trained with backpropagation using stochastic gradient descent with the Adam algorithm (Kingma and Ba, 2014) (learning rate = 10^–4^, β_1_ = 0.9, β_2_ = 0.99, CNN batch size = 128 epochs, LSTM batch size = 6 sleep periods) minimizing the sigmoid cross-entropy loss. For CNN, five recordings of the training set were held out as evaluation data for the early stopping criterion.

### Performance Evaluation

We used area under receiving operating characteristic curve (AUC) as the primary performance measure. The accuracy (ACC), sensitivity (Sens), specificity (Spes), positive predictive values (PPV) as well as confusion matrices were also calculated.

(1) $$A\,C\,C=\frac{T\,P+T\,N}{T\,P+T\,N+F\,P+F\,N}$$

(2) $$S\,e\,n\,s=\frac{T\,P}{T\,P+F\,N}$$

(3) $$S\,p\,e\,s=\frac{T\,N}{T\,N+F\,P}$$

(4) $$P\,P\,V=\frac{T\,P}{T\,P+F\,P}$$

where TP, TN, FP, and FN denote the numbers of true positives, true negatives, false positives and false negatives, respectively. A true positive is when a N3 epoch is correctly identified as N3. Further methodological description is provided in the online repository (Ranta, 2020).

## Results

Our final training dataset from the 43 infant PSG recordings included 21,913 epochs. Of these, 1,891 (8.6%) were excluded due to artifacts or absence periods. The remaining 20,022 epochs included 4,802 (24.0%) of N3 and 15,220 (76.0%) of other sleep stages (N1, N2, REM, and awake).

### Feature Selection

**FIGURE 2:**
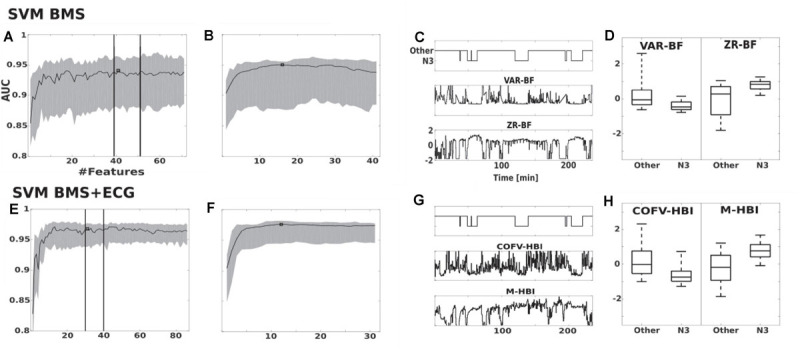
Feature selection of SVM BMS and SVM BMS + ECG classifiers. **(A,B)** The effect on AUC of adding features in the SVM BMS. The traces show the median and interquartile range of AUC in the two-step maximum-relevance-minimum-redundancy criteria feature selection **(A)** filter and **(B)** wrapper phase. Vertical lines indicate the consistent performance interval and square shows the selected feature set. **(C,D)** Examples of the two most important features, as a function of PSG study as well as comparison of all epochs. **(E–H)** Corresponding results for SVM BMS + ECG classifier. VAR-BF, variance in 6–16 Hz bed mattress signal band (BF), was the first selected feature for both SVM BMS and SVM BMS + ECG classifiers. The second selected feature was ZR-BF, the number of zero crossing of twice differentiated BF band, and COVF-HBI, the coefficient of variation of heart beat intervals (HBI), for SVM BMS and SVM BMS + ECG, respectively. The third selected feature was M-HBI, the mean of HBI for SVM BMS + ECG.

Individual features of the BMS and ECG signals are insufficient for the detection of N3 (see Figures 2C,D,G,H). Feature selection for the SVM BMS yielded 41 features in the first filter phase; the second wrapper phase resulted in 16 final features (Figures 2A,B). Feature selection for the SVM BMS + ECG yielded 31 features in the first phase and 12 features were selected in the second phase (Figures 2E,F). The selected features are listed in Table 3.

**TABLE 3:** Selected features for BMS and BMS + ECG-based SVMs.

**SVM BMS**	**SVM BMS + ECG**
1 **VAR-BF**	**VAR-BF**
2 ZR-BF*^d^*	COV-HBI
3 SE-BF	M-HBI
4 M-RF^†^	FUZE-HBI
5 **MOF**	LF/VLF
6 KURT-RCL	M2-BF
7 MOP	**MOF**
8 MO	LE-HBI
9 STD-BF*^a^	STD-RF^‡^
10 STD-BF*	HE-HBI
11 COMP-RF	**MOW**
12–16 KURT-BF **MOW** VAR-RF*^a^* KURT-RF*^a^* POW	KURT-BF*^a^*

*Each feature is indicated as a feature abbreviation and source signal-pair. Consensus features bolded. See Table 2 for abbreviations. ^*d*^Second difference, ^†^Extrema amplitude difference, ^‡^peaks, ^∗^Envelope, ^*a*^Activity event.*

### Classifier Performances

**FIGURE 3:**
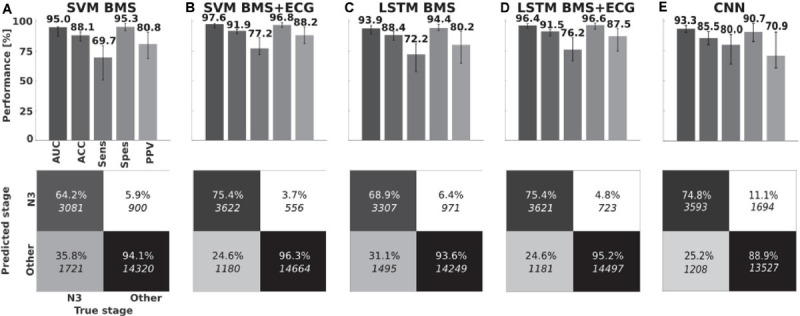
Classifier performances. Medians, interquartile ranges and pooled confusion matrices for **(A)** BMS feature-based Support Vector Machine classifier and **(B)** with ECG feature extension. **(C,D)** Long Short-Term Memory neural networks with same features. **(E)** Convolutional neural network on bed mattress signal. Area Under Receiving Operating Character Curve (AUC), accuracy (ACC), Sensitivity (Sens), Specificity (Spes), and positive predictive value (PPV).

The leave-one-patient-out cross-validation performance of SVM BMS over all 43 patients yielded a median AUC of 95.0% (IQR 87.7–95.6%). The SVM BMS with ECG derived features, had slightly increased performance with a median AUC of 97.6% (94.4–98.2%). Using a LSTM resulted in reduced performance (BMS features: median AUCs 93.9%, IQR 89.2–96.4%; BMS + ECG, median AUC 96.4%, IQR 94.2–98.2%. The CNN applied to the BMS signal resulted in a median AUC of 93.3% (IQR: 90.5–96.1%). However, there were no statistically significant differences in AUC between BMS classifiers (Friedman test, *p* = 0.977). Similarly, the Wilcoxon signed-rank test did not indicate a significant difference between BMS + ECG classifiers (*p* = 0.633). Additional results are in Figure 3 and Supplementary Table S3.

**FIGURE 4:**
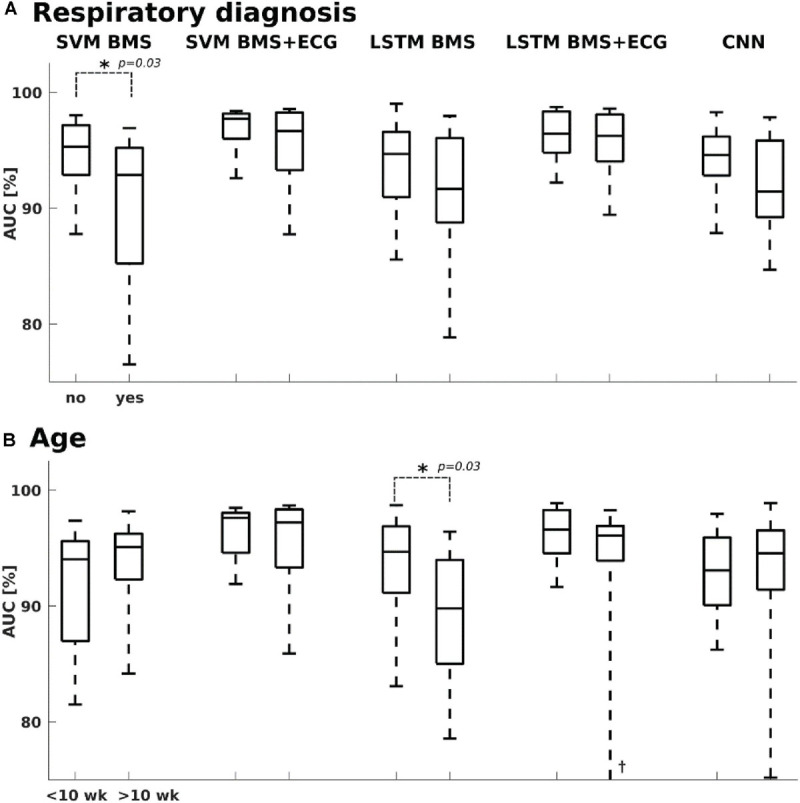
Classifier performance vs. clinical context. **(A)** SVM BMS classifier showed slightly higher performance with infants who did not have a diagnosis related to respiratory issues, while no significant difference was seen with other classifiers. **(B)** Only the performance of LSTM BMS was slightly lower with the older age group (> 10 weeks). ^†^Lower whisker at 58%. The age refers to postnatal age, or age after term equivalent age.

We also assessed the effect of clinical context on classifier performance (Figure 4), in particular presence of respiratory diagnoses (yes/no, *N* = 26/17) or infant’s age (under/above 10 weeks, *N* = 31/12). The accuracy of BMS-based classifiers was slightly affected by the clinical context: The SVM classifier performed a bit worse in infants with respiratory diagnoses (Mann-Whitney U test, *p* = 0.03 median AUC 92.9 vs. 95.3%), while the LSTM classifier showed somewhat better performance in younger infants (Mann-Whitney U-Test, *p* = 0.03; median AUC 94.6 vs. 89.8%). There was, however, no significant correlation between AUC and age (Spearman’s rank correlation; rho = −0.23 *p* = 0.14).

### Analysis of Features Selected for the Classifier

The most commonly selected features for BMS-based classifiers represent both cardiac and respiratory parameters. The variance of the ballistocardiogram pass-band (VAR-BF) reflects: (1) the reduced cardiac output which manifests the power of BF related to N3 sleep and (2) reduced gross-body movements during N3 which provides similar information to actigraphy. The number of zero crossings from the second difference of BF signal (ZR-BF) and BF spectral entropy (SE-BF) respond similarly. The mean difference of respiration band-pass peaks and troughs (M-RF^†^) measures the maximum momentary pressure changes in the BMS signal, and as such they are related to the depth of breathing movements. The respiration frequency is reduced in N3.

Feature selection yielded different feature sets on BMS + ECG classifiers, and only three features were in common with the list selected for BMS alone (Table 3). The ECG features selected focus on features describing heart rate variability (COV-HBI, FUZE-HBI, and LF/VLF) and the RR interval (M-HBI) (Figure 2F).

### Pilot Test in the NICU

**FIGURE 5:**
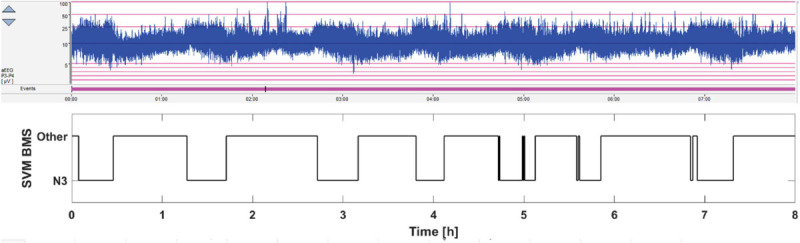
Pilot 8 h monitoring of sleep cycling in the NICU. The current standard practice is to observe SWC pattern in the aEEG trend (above), which refers to the alternation between thicker and thinner trends. The thicker aEEG trend comes from the intermittent, trace alternant/discontinue background pattern, which characterizes quiet (“deep”) sleep state. In contrast, the thinner aEEG trends come from the more continuous EEG pattern that characterizes the active sleep state. The BMS-based sleep monitoring is shown below. Note the faithful correspondence between thicker epochs in the aEEG trend and the deep sleep output of the BMS classifier.

Finally, we performed a recording on two newborn infants in the NICU to see how the BMS-based classifier would compare with the state-of-the-art visual inspection of the aEEG trend that is now routinely available at bedside. As shown in the Figure 5, there was a high correlation between the time course of BMS classifier and the “thickening” epochs in the aEEG trend that are known to correspond to quiet sleep. This correspondence between the “aEEG thickening” and the N3-detection is the ultimate aim for the BMS classifier as it will allow detection of cycling, or SWC. The results from our other pilot recording are shown in the Supplementary Figure S1, which demonstrated how the inherent ambiguity between sleep states may be seen in the dichotomic classifier output before post-processing. Some of the comparable ambiguity is also seen in Figure 5 (near 5 and 7 h). Much of that could be readily removed by post-processing, or by replacing the dichotomic classification with a sleep state probability index as shown before for a comparable EEG-based classifier (Koolen et al., 2017).

## Discussion

We show that an automated detection of deep sleep vs. other sleep states is feasible using fully non-invasive recording of BMS signal alone. We extend on previous work by providing an open source algorithm for infant sleep, which allows automated recognition of SWC. We show further with a pilot recording that such a method could be used as a real-time bedside trend of SWC in infants during intensive care (van den Hoogen et al., 2017) facilitating further development of clustered care and other procedures to minimize infants’ stress during care (van den Hoogen et al., 2017; Knauert et al., 2018). Measuring the amount of deep/quiet sleep (Shellhaas et al., 2017) or the cycling between sleep states (Thoresen et al., 2010; Klebermass et al., 2011) have prognostic value as they are associated with neurodevelopmental outcomes.

The feature selection protocol showed that a reasonable classification accuracy can be achieved with only a handful of features, while the majority of the examined 71 computational features are essentially unhelpful or redundant. This demonstrates that smaller feature sets could be sufficient if computational complexity needs to be optimized, e.g., for an online algorithm or embedded implementation. The addition of features derived from the ECG heart beat increased classifier performance; however, it comes at the cost of needing a merger of BMS and ECG sensor signals.

The two BMS classifier designs, one based on SVM and the other based on LSTM, demonstrated a comparable performance in identifying N3 sleep stage at the full cohort level. However, the SVM-based classifier showed more variability between subjects, and the LSTM-based classifier showed a better performance with the younger subjects. A respiratory diagnosis lowered performance of the classifier. This could be due to pathophysiological changes in breathing stability. Comparable performance between CNN and SVM classifiers indicates that the feature representation can be learned directly from the BMS signal.

Our findings are in agreement with findings from prior studies in infants (Thoman and Glazier, 1987; Sazonova et al., 2006; Terrill et al., 2012; Isler et al., 2016). Thoman and Glazier (1987) reported the total accuracy of 80.6% (active, quiet, transition, awake) for their BMS respiration and gross-body movement-based classifier. However, they used a small sample size (*n* = 10) and their in-house behavioral scoring criteria as a reference (Thoman, 1975). Deploying different source signals for respiration and gross-body movements has yielded variable though lower (53–70%) accuracies in distinguishing active and quiet sleep states from the RIP or actigraphy signals (*N* = 26) (Sazonova et al., 2006). Terrill et al. developed a novel computational measure based on recurrence quantification analysis of RIP signals, which allowed sleep classification at a significantly higher accuracy (80%) (Terrill et al., 2012). Likewise, the variance of instantaneous breathing rate was shown to yield over 80% accuracy (Isler et al., 2016). An exact comparison of classifier algorithms between our study and the earlier works is challenging because we used an updated sleep scoring reference (Grigg-Damberger et al., 2007), and there are poorly translatable differences between the physiological signals used in different studies. Our fully non-obtrusive BMS signal might be more sensitive to, e.g., movement artifacts than the body-attached RIP sensor used in prior studies (e.g., Terrill et al., 2012). However, our presently introduced classifier is in a general agreement with the prior works.

The presently introduced BMS-based detector has a limited focus: It aims to detect quiet sleep only. It is not aimed to provide a full multiclass classification of all sleep states, neither does it allow diagnostic measures of specific sleep–related adversities. Such in-depth sleep analyses would require more comprehensive measures of physiological parameters which are routinely available in the existing PSG paradigms. Moreover, it will be important to validate the use of our BMS-based classifier in different kinds of user scenarios in the NICU, including, e.g., infants born preterm, undergoing ventilatory support, or receiving sedative medication or hypothermia therapy; all these factors may have different effects on the relationship between respiration and sleep states. These prospective studies are on-going, and they will become possible in many independent centers with the open-access algorithm provided in this work.

The common challenge with sleep classifiers is in detecting the exact timing of sleep state transition, which lowers the nominal performance. This is also a challenge in clinicians’ visual scoring, and it may substantially lower the inter-rater agreement (Satomaa et al., 2016). Notably, visual scoring of PSG studies relies heavily on multiple physiological signals, including cortical activity from the EEG, while our presently developed classifier relies on respiratory and cardiac activity alone. Adding the other PSG signals to the classifier would undoubtedly improve classifier performance, however it would also directly affect the utility of the classifier as a non-invasive measure for longer term monitoring. It is crucial in this context to consider the aimed use of the automated classifier of this kind. The ultimate purpose is to estimate cycling/fluctuation of vigilance states rather than to estimate accurate transition times (SWC, Kidokoro et al., 2012; van den Hoogen et al., 2017). This implies that identifying any sleep state, such as N3 in this work, with reasonable accuracy would allow quantification of SWC (Stevenson et al., 2014), or its fragmentation. This deflates the importance of exact transition times as long as the overall pattern is detected with a reasonable accuracy.

## Conclusion

We introduce an automated signal processing pipeline for infant deep sleep detection from the BMS signal with or without ECG signal. The proposed method allows long-term monitoring of sleep-wake cycling, a key bedside index in the NICU brain monitoring. We also provide pilot proof of concept evidence that this closely corresponds to the SWC observed in the currently available aEEG-based review, hence the method can be applied in the NICU to allow future development of treatments and nursing practices with a minimally disturbed sleep (van den Hoogen et al., 2017).

## Data Availability Statement

The original contributions presented in the study are included in the article/Supplementary Material, further inquiries can be directed to the corresponding author/s.

## Ethics Statement

The studies involving human participants were reviewed and approved by the Institutional Review Ethics Committee of Helsinki Hospital, Helsinki, Finland. Written informed consent from the participants’ legal guardian/next of kin was not required to participate in this study in accordance with the national legislation and the institutional requirements.

## Author Contributions

JR developed the support vector machine and Long Short-Term Memory neural network infant sleep classifiers, conducted sleep classifier evaluation, and comparison and contributed writing the article. MA developed the convolutional neural network classifier and contributed writing the article. TK conducted the infant polysomnygraphy and sleep mattress data collection in addition to sleep stage scoring. SV coordinated the project, contributed clinical, and physiological knowledge on designing the classifiers and took part in writing. NS coordinated the project, took part in designing the classifiers, and contributed writing the article. All authors contributed to the article and approved the submitted version.

## Conflict of Interest

JR was a shareholder and a part time employee in sensor manufacturer Emfit Ltd. Emfit did not have any role in study design, data analysis or publication process. This work was mostly done before JR was employed by Emfit. The remaining authors declare that the research was conducted in the absence of any commercial or financial relationships that could be construed as a potential conflict of interest.
