# Supplementary material for: An Open Source Classifier for Bed Mattress Signal in Infant Sleep Monitoring
Source: Front Neurosci. 2021 Jan 14;14:602852. doi: 10.3389/fnins.2020.602852 (PMC7840576; doi:10.3389/fnins.2020.602852)

**Supplementary material**

*Ranta et al. An open-source classifier for bed mattress signal in infant sleep studies*

**Table S1:** Summary of clinical information in each patient.

|  | **age at recording** | **Clinical background** |  |  |
| --- | --- | --- | --- | --- |
| **Study #** | **(days)** | **preterm, asphyxia, other** | **PSG indication** | **PSG finding** |
| 2 | 56 | Robin sdr. | Robin | Mild OSA |
| 3 | 35 | preterm 35+5 | Apnea | Mild CSA |
| 4 | 73 |  | ALTE | OSA |
| 6 | 125 | preterm 36+2, Chiari I | Chiari I | Normal |
| 8 | 42 |  | ALTE | Normal |
| 9 | 35 | Brain MRI, abnormal | Apnea | Mild OSA |
| 10 | 31 |  | SIDS sibling | Normal |
| 11 | 46 | Robin sdr. | Robin | Mild OSA |
| 13 | 12 |  | Apnea | Mild OSA |
| 14 | 40 |  | Previous CSA | Normal |
| 15 | 13 |  | Central hypopnea | CSA+hypoventilation |
| 16 | 70 | Mild asphyxia | Apnea | Mild OSA |
| 17 | 19 | Achondroplacia | narrow foramen magnum | Normal |
| 18 | 108 | hypotonia nas | Hypotonia | OSA + increased WOB |
| 19 | 122 | preterm 26+2 | Apnea | Normal |
| 20 | 90 | preterm 35+5 | CSA | CSA |
| 21 | 76 | Brain MRI, abnormal | Apnea | Normal |
| 22 | 98 | Erb's paresis | suspicion of hypoventilation | Normal |
| 23 | 35 |  | central hypoventilation, treated | Severe CSA, mild hypoventilation |
| 24 | 35 |  | OSA | Normal |
| 25 | 30 |  | ALTE | Normal |
| 26 | 25 | Robin sdr. | Robin | Mildly increased WOB |
| 27 | 35 | Robin sdr. | Robin | OSA |
| 28 | 118 | preterm 26+1, w/IVH | Stridor | Increased breathing effort |
| 29 | 42 | Robin sdr. | Robin | OSA |
| 30 | 43 | Robin sdr. | Robin | Mild OSA |
| 31 | 81 |  | Obstructed airway | OSA + increased WOB |
| 32 | 127 | preterm 35+6, MRI abnormalities | Abnormal corpus callosum | Mildly increased WOB |
| 34 | 78 | Achondroplacia | Achondroplacia | Normal |
| 35 | 82 | Robin sdr. | Robin | Normal/mild OSA |
| 36 | 27 | Robin sdr. | Robin | OSA |
| 37 | 66 | preterm 32+2 | ALTE | OSA |
| 38 | 42 | Robin sdr. | Robin | Normal |
| 39 | 54 |  | Abnormal breathing | Normal |
| 40 | 44 |  | Abnormal breathing | Mildly increased WOB |
| 41 | 24 | Robin sdr. | Robin | Normal |
| 42 | 19 |  | CSA | Normal |
| 43 | 16 | bone dysplasia nas | Apnea | Normal |
| 44 | 14 | Robin sdr. | Robin | OSA |
| 46 | 24 |  | Small chin | Mild OSA |
| 49 | 59 | preterm 36+6 | Apnea | OSA |
| 50 | 21 | Robin sdr. | Robin | Mild OSA |
| 51 | 36 | preterm 35+5 | Abnormal breathing | Normal |

**Table S2:** Network architecture of the utilized CNN model. The *input module* of the CNN performs low-level feature extraction from each frame, and finally compresses each frame into a 16-dimensional representation. The *Temporal module* contains five stacked residual modules, which model temporal (frame-to-frame) dynamics with gated dilated convolutions (van den Oord et al. 2016). The *Postnet module* finally transforms the representation into the binary decision. *N* denotes the number of 30-second frames in a processed recording.

|  | Operation | Details | Input size | Output size |
| --- | --- | --- | --- | --- |
| *Input module:* Layer 1: | Conv1D | act=tanh, fw=240, stride=120, chans=16 | (*N*,6000,1) | (*N*,50,16) |
| Layer 2: | Conv1D | act=ReLU, fw=5, stride=2, chans=16 | (*N*,50,16) | (*N*,25,16) |
| Layer 3: | Flatten | - | (*N*,25,16) | (*N*,400) |
| Layer 4: | Dense | act=ReLU, dims=16 | (*N*,400) | (*N*,16) |
| *Temporal module:* Layers 5-9: | Residual module | fw=3, dilations=[1,2,4,8,16] | (1,*N*,16) | (1,*N*,16) |
| *Postnet module:* Layer 10: | Conv1D | act=ReLU, fw=3, stride=1, chans=16 | (1,*N*,16) | (1,*N*,16) |
| Layer 11: | Dense | act=Sigmoid, dims=1 | (*N*,16) | (*N*,1) |

**Table S3:** Comparison of feature-based Support Vector Machine and Long Short-Term Memory classifier performances to convolutional neural network sleep classifier. Patient median (interquartile ranges) for Area Under Receiving Operating Character Curve (AUC), accuracy (ACC), Sensitivity (Sens), Specificity (Spes) and positive predictive value (PPV).

|  | AUC (%) | ACC (%) | Sens (%) | Spes (%) | PPV (%) |
| --- | --- | --- | --- | --- | --- |
| **SVMBMS** | 95.0 [87.7; 95.6] | 88.1 [83.4; 91.1] | 69.7 [50.9; 79.3] | 95.3 [92.3; 98.1] | 80.8 [68.8; 91.0] |
| **SVMBMS+ECG** | 97.6 [94.4; 98.2] | 91.9 [89.6; 93.6] | 77.2 [72.1; 86.3] | 96.8 [95.0; 98.7] | 88.2 [81.2; 95.2] |
| **LSTMBMS** | 93.9 [89.2; 96.4] | 88.4 [84.2; 91.3] | 72.2 [57.7; 81.1] | 94.4 [92.4; 97.5] | 80.2 [65.0; 91.5] |
| **LSTMBMS+ECG** | 96.4 [94.2; 98.2] | 91.5 [87.7; 93.2] | 76.2 [66.8; 86.4] | 96.6 [93.5; 99.4] | 87.5 [75.1; 93.9] |
| **CNN** | 93.3 [90.5; 96.1] | 85.5 [80.2; 91.0] | 80.0 [64.1; 88.4] | 90.7 [82.8; 97.8] | 70.9 [60.7; 90.7] |

**Figure S1**

The second infant with pilot 4 hours monitoring of sleep cycling in the NICU. The quiet/deep sleep periods are seen as thicker aEEG trend (marked with green bars), which is consistently reflected as a longer period of N3 detection by the SVM algorithm (based on BMS alone). Notably, this infant has less matured sleep cycling, and there is consequently some ambiguity in the SVM-detection, seen as the brief switches between other vs N3 predictions. Some of such ambiguity could be removed by post-processing of the detections with e.g. smoothing, while some of the ambiguity is likely real and should be reflected in the SVM output as well. For further details, please see Figure 5 caption.


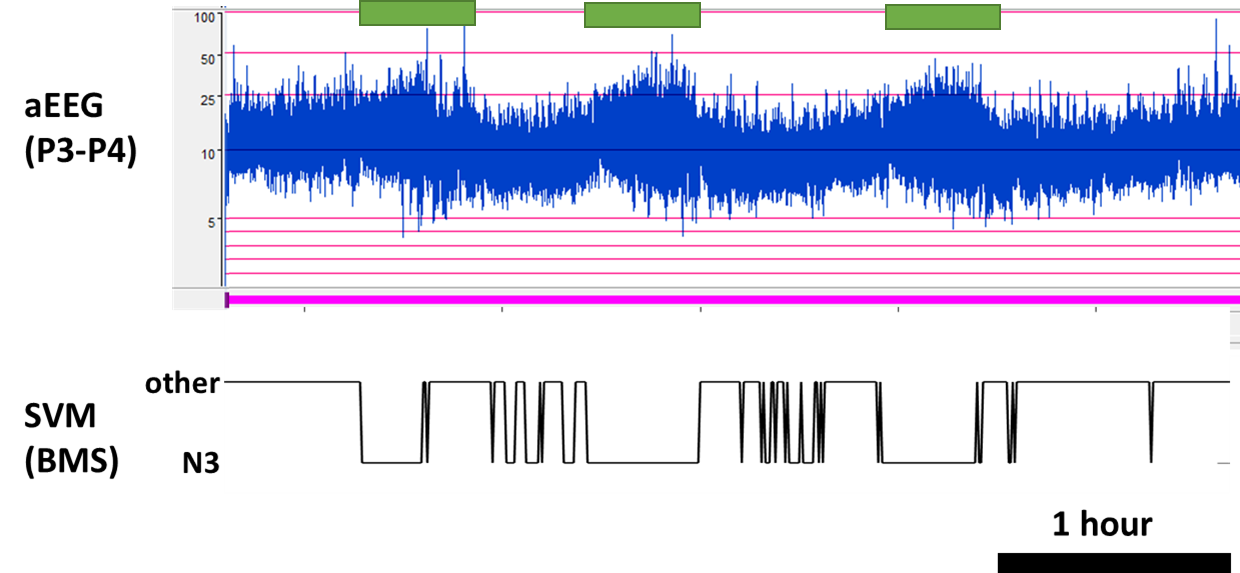

Supplement: Supplementary file 1 [file Data_Sheet_1.docx]
